# Add-on mepolizumab in patients with severe asthma on medium-dose inhaled corticosteroids

**DOI:** 10.1183/23120541.01652-2025

**Published:** 2026-08-17

**Authors:** Ruchong Chen, Liping Wei, Meiling Jin, Yi Jiang, Xinglin Gao, Li Zhao, Limin Wang, Shengming Liu, Peter Howarth, Cui Xiong, Jie Huang, Peiwen Liang, Xiao Hu, Nanshan Zhong

**Affiliations:** 1 State Key Laboratory of Respiratory Disease, Joint International Research Laboratory of Respiratory Health, National Clinical Research Center for Respiratory Disease, Guangzhou National Laboratory, Department of Allergy and Clinical Immunology, Guangzhou Institute of Respiratory Health, The First Affiliated Hospital of Guangzhou Medical University, Guangzhou, China; 2 Department of Pulmonary and Critical Care Medicine, 3rd Affiliated Hospital of Guangzhou, Guangzhou, China; 3 Department of Allergy and Clinical Immunology, Zhongshan Hospital Fudan University, Shanghai, China; 4 Department of Pulmonary and Critical Care Medicine, The First Hospital of Shanxi Medical University, Taiyuan, China; 5 Department of Pulmonary and Critical Care Medicine, Guangdong Provincial People's Hospital, Guangdong Academy of Medical Sciences, Southern Medical University, Guangzhou, China; 6 Department of Pulmonary and Critical Care Medicine, Shengjing Hospital of China Medical University, Shenyang, China; 7 Department of Pulmonary and Critical Care Medicine, Hangzhou First People's Hospital, Hangzhou, China; 8 Department of Pulmonary and Critical Care Medicine, The First Affiliated Hospital of Jinan University, Guangzhou, China; 9 Global Medical Affairs, Specialty Medicine, GSK, Brentford, UK; 10 R&D, GSK, Shanghai, China; 11 Medical Affairs, GSK, Shanghai, China; 12 R.Chen, L. Wei and M. Jin contributed equally to this work

## Abstract

**Background:**

Mepolizumab is clinically beneficial for patients with severe asthma with an eosinophilic phenotype (SA-EP) but lacks randomised controlled trial efficacy data in those on medium-dose inhaled corticosteroids (ICS). This analysis assessed mepolizumab's efficacy in a subgroup of patients on medium-dose ICS from a Chinese phase 3 trial (NCT03562195).

**Methods:**

Eligible SA-EP patients had received daily ≥500 µg·day^−1^ fluticasone propionate or equivalent. In this *post hoc* evaluation, we included patients who were on baseline medium-dose ICS. Outcomes assessed were rate of clinically significant exacerbations (CSEs) at week 52, time to first CSE, St George's Respiratory Questionnaire (SGRQ) score, pre-bronchodilator forced expiratory volume in 1 s (FEV_1_), asthma control questionnaire-5 (ACQ-5) score and rate of clinical remission.

**Results:**

At week 52, in the medium-dose ICS subgroup, mepolizumab (n=115) *versus* placebo (n=104) significantly reduced the rate of CSEs (0.53 *versus* 1.30 events·yr^−1^; rate ratio 0.41 (95% CI 0.25–0.66); p<0.001), with a lower probability of CSE (Kaplan–Meier estimate, 27.0% *versus* 49.3%; HR 0.47 (95% CI 0.30–0.73); p<0.001), greater improvements, as measured by difference in least-square mean, in SGRQ score (−5.58, 95% CI −10.73 to −0.44), FEV_1_ (119.06 mL, 95% CI 6.66–231.45) and ACQ-5 score (−0.25, 95% CI −0.46 to −0.04), and a higher proportion of patients achieving three- or four-component clinical remission (31.3–57.4% *versus* 13.5–36.5%). The rate of clinical remission was higher in patients on medium-dose ICS than those on high-dose ICS.

**Conclusions:**

Mepolizumab provided greater clinical benefit across multiple outcomes including clinical remission *versus* placebo in Chinese SA-EP patients on medium-dose ICS. Earlier treatment could increase the chance of clinical remission.

## Shareable abstract

**Add-on mepolizumab reduced clinically significant exacerbations, increased health-related quality of life and lung function, and induced clinical remission *versus* placebo in patients with severe asthma on medium-dose inhaled corticosteroids** https://bit.ly/3OWjoAc

## Introduction

Severe asthma is defined as asthma that requires treatment with medium to high-dose inhaled corticosteroids (ICS) plus a second controller and/or systemic corticosteroids (*e.g.* oral corticosteroid (OCS)), to prevent it from becoming “uncontrolled” or that remains “uncontrolled” despite this therapy [1–3]. It is associated with frequent exacerbations, poor disease control, impaired quality of life, damaged lung function and fixed airflow obstruction [3–6]. Additionally, the condition contributes to considerable morbidity and mortality and demands substantial healthcare resources [3, 7, 8]. The eosinophilic phenotype, characterised by a high number of eosinophils in sputum and peripheral blood [9, 10], comprises >75% of severe asthma cases [11–13]. Patients with this phenotype tend to experience more asthma attacks, have more comorbidities and greater healthcare resource use (HCRU) [14]. There remains a critical need to effectively manage severe asthma with an eosinophilic phenotype (SA-EP).

For patients whose asthma remains uncontrolled despite optimised treatment with medium-dose ICS, treatment escalation from medium-dose to high-dose ICS is recommended, but only for short-term use [1, 15]. The use of high-dose ICS, however, faces several limitations. First, the increase in ICS dose generally provides little additional benefit [15]. For instance, in patients with moderate to severe asthma, fluticasone propionate (FP) achieves most of the clinical benefits at low-dose levels [16], while the risk of side effects increases with higher doses of ICS, depending upon their systemic bioavailability [15–18]. These side effects include systemic and local side effects, such as adrenal suppression, cataracts, osteoporosis, fractures, diabetes, oral candidiasis and dysphonia, imposing a high burden on patients [15, 16, 18].

Moreover, although ICS can effectively control asthma symptoms, they do not significantly impact the long-term course of the disease [3, 19, 20]. This limitation becomes particularly pertinent as asthma management increasingly focuses on clinical remission, which is broadly defined as sustained symptom control, elimination of OCS exposure and exacerbations and stabilisation of pulmonary function [3]. Recognising this, the Global Initiative for Asthma (GINA) has included clinical remission in its recent report, emphasising that it aligns with the long-term treatment goals of asthma [15]. This evolving focus underscores the need for asthma therapies that can fundamentally modify the disease's course [3]. Thus, there is an urgent need for more effective and safer treatment options that can be employed early in the treatment pathway to address the shortcomings of high-dose ICS and potentially help patients achieve clinical remission.

Biologics are recommended as an add-on therapy for SA-EP patients whose asthma cannot be well controlled by ICS-based standard therapy alone [15]. The use of these targeted therapies has made clinical remission an attainable treatment goal. Mepolizumab, a humanised monoclonal antibody that targets interleukin (IL)-5, is approved for SA-EP in several regions, including China [21–23]. Studies have consistently demonstrated that mepolizumab, which is generally well tolerated, can reduce exacerbations and OCS use, improve asthma control, lung function and health-related quality of life, and induce clinical remission in patients with SA-EP [24–30]. Given its favourable efficacy and safety profiles, mepolizumab as an alternative to high-dose ICS warrants exploration. The real-world REALITI-A study has demonstrated that in SA-EP patients on medium-dose ICS at baseline, mepolizumab treatment reduced the rate of clinically significant exacerbations (CSEs) and median daily dose of maintenance ICS, compared with the pre-treatment period [31]. However, to date, this is the only analysis of mepolizumab in patients on medium-dose ICS. Additionally, no studies have examined the treatment effect of mepolizumab *versus* a comparator in this patient population.

To further understand the efficacy of mepolizumab in SA-EP patients on medium-dose ICS, we conducted a *post hoc* subgroup analysis of a phase III, randomised, double-blind, placebo-controlled trial (the 201536 study, NCT03562195) of mepolizumab in Chinese patients with SA-EP [32]. The study demonstrated that mepolizumab as add-on treatment reduced CSEs, increased health-related quality of life and lung function and induced clinical remission compared with placebo in Chinese SA-EP patients [25, 32]. The current analysis based on randomised clinical trial (RCT) data focuses on patients on medium-dose ICS at baseline, representing the first attempt to explore the treatment effect of mepolizumab *versus* a comparator in SA-EP patients on medium-dose ICS.

## Methods

### Study design and participants

The 201536 study (clinicaltrials.gov, NCT03562195, registered on 22 May 2018) was a phase III, randomised, double-blind, placebo-controlled, parallel-group study conducted across 45 sites in China from August 2018 to September 2022. The study was performed in accordance with the principles of the Declaration of Helsinki and all mandatory safety procedures were complied with in conducting the experimental work. The ethics committee of each participating centre approved the study. All patients provided their written informed consent. The details of the study have been reported elsewhere [32].

Eligible patients were ≥12 years of age, weighed ≥40 kg and had asthma with an eosinophilic phenotype (peripheral blood eosinophil count of ≥150 cells·µL^−1^ at screening or ≥300 cells·µL^−1^ during the previous 12 months) and persistent airflow obstruction at the baseline visit. Prior to the baseline visit, patients had received regular treatment with ICS (≥500 µg·day^−1^ FP or equivalent) for 12 months in total (with the last 3 months immediately preceding the baseline visit), with or without maintenance OCS (≥5 mg·day^−1^ prednisone or equivalent), and ≥1 additional controller medication for ≥3 months. Patients experienced ≥2 exacerbations requiring systemic corticosteroids in the previous 12 months. Patients were randomised 1:1 to receive mepolizumab 100 mg or placebo subcutaneously every 4 weeks for 52 weeks.

This *post hoc* subgroup analysis focused on patients on medium-dose ICS at baseline. The definitions for “medium-dose” and “high-dose” ICS were consistent with those in the GINA report and the 2020 Chinese guidelines [1, 15]. Namely, medium-dose ICS was defined as >250 and ≤500 µg·day^−1^ of FP or its equivalent, and high-dose ICS >500 µg·day^−1^ of FP or its equivalent.

### End-points

The primary end-point was the annualised rate of CSEs over the 52-week treatment period. CSE was defined as worsening of asthma that required the use of systemic corticosteroids and/or hospitalisations and/or emergency department (ED) visits. Secondary end-points included time to first CSE and changes from baseline in St George's Respiratory Questionnaire (SGRQ) score and pre-bronchodilator forced expiratory volume in 1 s (FEV_1_) at week 52. Other end-points included change from baseline in asthma control questionnaire-5 (ACQ-5) score at week 52 and time to first exacerbation requiring hospitalisation or ED visits.

This *post hoc* analysis explored clinical remission at week 52, using both three- and four-component definitions. The three-component definition consisted of 1) no maintenance OCS use for the entire 90-day period prior to week 52; 2) no CSE during the 52 weeks of treatment; and 3) an ACQ-5 score of <1.5 or ≤0.75 at week 52. An additional criterion was added to the four-component definition: a change from baseline in pre-bronchodilator FEV_1_ of ≥0 mL over 52 weeks.

### Statistical analysis

Patients with missing ICS dose data at baseline were excluded from the current analysis. All pre-specified end-points were analysed using the same approaches as in the primary analysis, except that treatment was the only covariate used [32]. The mepolizumab and placebo groups were compared in terms of the proportion of patients who achieved clinical remission, using logistic regression with treatment arm (mepolizumab or placebo) as the only covariate. All p-values reported were two-sided and nominal. For a continuous variable, mean ±sd are reported. For a categorical variable, the number and percentage for each category are reported. All programming for statistical analyses was performed using SAS v.9.4 or later.

## Results

Of 371 patients screened, 300 were randomised, with 149 in the mepolizumab group and 151 in the placebo group (supplementary figure S1). At baseline, 219 (73%) were receiving medium-dose ICS (115 mepolizumab, 104 placebo) and 72 (24%) were receiving high-dose ICS (31 mepolizumab, 41 placebo). Baseline characteristics for patients are presented in table 1. For patients on medium-dose ICS at baseline, the mean age was 51.9±12.8 years, the mean duration of asthma was 11.2±11.0 years, the mean number of exacerbations in the 12 months prior to screening was 2.8±1.46, and 14 (6.4%) patients were on maintenance OCS, with a mean dose of 8.3±4.4 mg·day^−1^ (table 1).

**TABLE 1:** Baseline characteristics of patients on medium- and high-dose ICS at baseline

	Medium-dose ICS at baseline			High-dose ICS at baseline		
	Placebo (n=104)	Mepolizumab 100 mg *s.c.* (n=115)	Total (n=219)	Placebo (n=41)	Mepolizumab 100 mg *s.c.* (n=31)	Total (n=72)
**Age, y**	53.7±12.92	50.3±12.58	51.9±12.82	53.0±15.81	52.4±12.64	52.7±14.44
<65	81 (77.9)	106 (92.2)	187 (85.4)	31 (75.6)	26 (83.9)	57 (79.2)
≥65	23 (22.1)	9 (7.8)	32 (14.6)	10 (24.4)	5 (16.1)	15 (20.8)
**Sex**						
Female	61 (58.7)	64 (55.7)	112 (57.1)	19 (46.3)	16 (51.6)	35 (48.6)
**Race**						
Asian	104 (100)	115 (100)	219 (100)	41 (100)	31 (100)	72 (100)
**Weight, kg**	62.9±11.79	66.9±12.57	65.0±12.35	68.1±13.47	64.4±12.32	66.5±13.03
**BMI, kg·m^−2^**	24.0±3.80	25.0±3.86	24.5±3.85	24.9±3.70	24.5±3.77	24.7±3.71
**Duration of asthma, y**	11.6±11.44	10.7±10.59	11.2±10.98	13.6±11.65	13.5±11.20	13.6±11.37
**Baseline lung function**						
Pre-bronchodilator FEV_1_, mL	1513±641	1633±694	1576±671	1590±791	1533±470	1565±668
Post-bronchodilator FEV_1_, mL	1682±641	1846±717	1768±686	1786±793	1734±534	1764±690
Pre-bronchodilator FVC, mL	2570±874	2828±894	2706±892	2744±1025	2558±704	2664±900
Pre-bronchodilator FEV_1_:FVC ratio	0.58±0.12	0.57±0.12	0.57±0.12	0.57±0.14	0.61±0.12	0.58±0.13
FEV_1_ reversibility, mL	267±200	274±198	271±199	271±172	267±170	269±170
**Total number of exacerbations in the past 12 months prior to screening**						
2	65 (62.5)	73 (63.5)	138 (63.0)	17 (41.5)	15 (48.4)	32 (44.4)
3	23 (22.1)	20 (17.4)	43 (19.6)	11 (26.8)	9 (29.0)	20 (27.8)
4	9 (8.7)	11 (9.6)	20 (9.1)	6 (14.6)	4 (12.9)	10 (13.9)
>4	7 (6.7)	11 (9.6)	18 (8.2)	7 (17.1)	3 (9.7)	10 (13.9)
Number of exacerbations	2.7±1.32	2.8±1.57	2.8±1.46	3.1±1.23	2.9±1.19	3.0±1.21
**Current medical conditions^#^**						
Allergic rhinitis or hay fever	53 (51.0)	62 (53.9)	115 (52.5)	24 (58.5)	18 (58.1)	42 (58.3)
Hypertension	27 (26.0)	36 (31.3)	63 (28.8)	10 (24.4)	12 (38.7)	22 (30.6)
Sinusitis	15 (14.4)	21 (18.3)	36 (16.4)	5 (12.2)	6 (19.4)	11 (15.3)
Coronary artery disease	11 (10.6)	8 (7.0)	19 (8.7)	7 (17.1)	4 (12.9)	11 (15.3)
Diabetes mellitus	14 (13.5)	8 (7.0)	22 (10.0)	5 (12.2)	1 (3.2)	6 (8.3)
History of anaphylaxis	9 (8.7)	10 (8.7)	19 (8.7)	5 (12.2)	3 (9.7)	8 (11.1)
Arrhythmia	5 (4.8)	6 (5.2)	11 (5.0)	6 (14.6)	0	6 (8.3)
**Blood eosinophil count ≥150 cells·μL^−1^ that was related to asthma at screening**	90 (86.5)	97 (84.3)	187 (85.4)	37 (90.2)	27 (87.1)	64 (88.9)
**Blood eosinophil count ≥300 cells·μL^−1^ that was related to asthma in the past 12** **months prior to screening**	88 (84.6)	98 (85.2)	186 (84.9)	36 (87.8)	27 (87.1)	63 (87.5)
**Baseline OCS daily dose, n**	8 (7.7)	6 (5.2)	14 (6.4)	3 (7.3)	3 (9.7)	6 (8.3)
<7.5 mg·day^−1^	3 (37.5)	3 (50.0)	6 (42.9)	1 (33.3)	2 (66.7)	3 (50.0)
≥7.5 and <15 mg·day^−1^	4 (50.0)	2 (33.3)	6 (42.9)	1 (33.3)	1 (33.3)	2 (33.3)
≥15 and <30 mg·day^−1^	1 (12.5)	1 (16.7)	2 (14.3)	1 (33.3)	0	1 (16.7)
≥30 mg·day^−1^	0	0	0	0	0	0
Daily dose, mg·day^−1^	8.4±4.8	8.2±4.3	8.3±4.4	13.3±10.4	6.7±2.9	10.0±7.8

Data are presented as mean±sd or n (%). BMI: body mass index; FEV_1_: forced expiratory volume in 1 s; FVC: forced vital capacity; ICS: inhaled corticosteroid; OCS: oral corticosteroids. ^#^: Current medical conditions present in ≥10% of patients on either medium- or high-dose ICS at baseline are shown.

**FIGURE 1:**
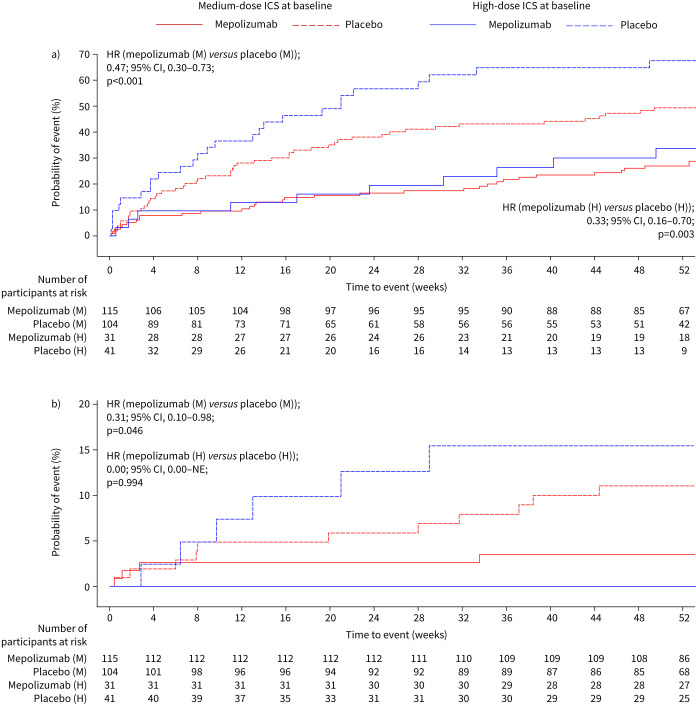
Kaplan–Meier cumulative incidence curves for a) time to first clinically significant exacerbation, and b) time to first exacerbation requiring hospitalisation or emergency department (ED) visits in patients on medium-dose or high-dose inhaled corticosteroids (ICS) at baseline. HR: hazard ratio; NE: not estimable.

At week 52, in patients on medium-dose ICS at baseline, the rate of CSEs in the mepolizumab arm was 0.53 events·yr^−1^, significantly lower than 1.30 events·yr^−1^ in the placebo arm (rate ratio 0.41, 95% CI 0.25–0.66; p<0.001). Time to first CSE by week 52 was significantly longer in the mepolizumab arm than in the placebo arm. According to the Kaplan–Meier estimates, the probability of experiencing a CSE by week 52 was 27.0% in the mepolizumab group, significantly lower than 49.3% in the placebo group (HR 0.47, 95% CI 0.30–0.73; p<0.001) (figure 1a). Similarly, time to first exacerbation requiring hospitalisation or ED visits by week 52 was significantly longer in the mepolizumab arm than in the placebo arm, with Kaplan–Meier estimates indicating a 3.5% probability of experiencing such an exacerbation in the mepolizumab arm *versus* 11.0% in the placebo arm (HR 0.31, 95% CI 0.10–0.98; p=0.046) (figure 1b). In patients on high-dose ICS at baseline, similar clinical benefits in terms of reduction in CSE and a lower probability of CSE were observed with mepolizumab *versus* placebo (figure 1 and supplementary table S1).

**FIGURE 2:**
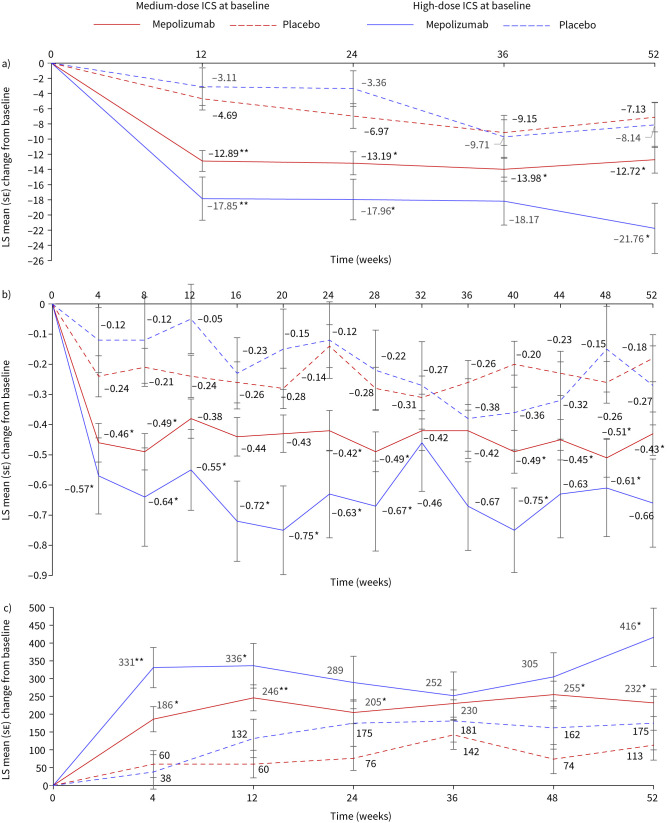
Least-square (LS) mean changes from baseline in a) St George's Respiratory Questionnaire score, b) asthma control questionnaire-5 score and c) pre-bronchodilator forced expiratory volume in 1 s in patients on medium-dose or high-dose inhaled corticosteroids (ICS) at baseline. *: p<0.05; **: p<0.001.

Throughout the 52-week treatment period, greater improvements in the SGRQ and ACQ-5 scores, as indicated by greater decreases in their least-square means from baseline, were consistently seen with mepolizumab *versus* placebo in the medium-dose ICS subgroup (figure 2a,b). Significantly greater improvements in SGRQ and ACQ-5 scores were observed with mepolizumab *versus* placebo as early as the first assessment (week 12 and week 4, respectively) (figure 2a,b). Notably, the improvement in SGRQ score remained significant at all subsequent time points through week 52. Similarly, greater improvement in FEV_1_, as indicated by greater increases in its least-square means from baseline, was observed with mepolizumab *versus* placebo throughout the treatment period (figure 2c). Significantly greater improvements in FEV_1_ were observed with mepolizumab *versus* placebo at as early as week 4 (figure 2c). At week 52, the difference in least-square mean between mepolizumab and placebo arms was −5.58 (95% CI −10.73 to −0.44; p=0.034) for the SGRQ score, −0.25 (95% CI −0.46 to −0.04; p=0.019) for the ACQ-5 score and 119.06 mL (95% CI 6.66–231.45; p=0.038) for FEV_1_. In patients on high-dose ICS at baseline, similar clinical benefits in terms of improvements in SGRQ score, ACQ-5 score and FEV_1_ were observed with mepolizumab *versus* placebo (figure 2 and supplementary table S1).

**FIGURE 3:**
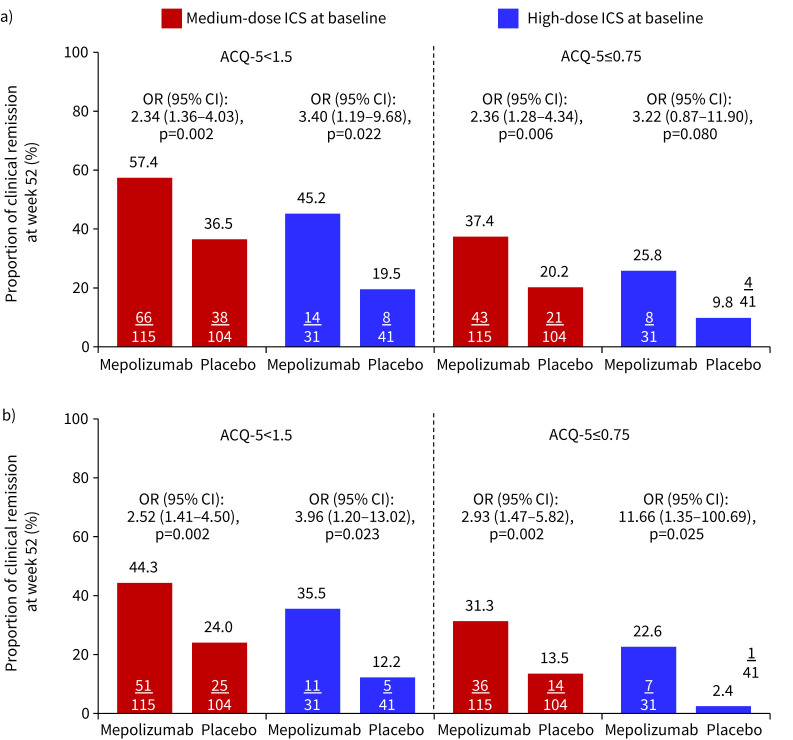
Proportions of patients who achieved clinical remission at week 52 according to a) three-component definition and b) four-component definition in patients on medium-dose or high-dose inhaled corticosteroids (ICS) at baseline. ACQ-5: asthma control questionnaire-5; OR: odds ratio.

With an ACQ-5 score <1.5, 57.4% (66 of 115) of the mepolizumab arm and 36.5% (38 of 104) of the placebo arm met the three-component definition of clinical remission (OR 2.34, 95% CI 1.36–4.03; p=0.002). With an ACQ-5 score ≤0.75, 37.4% (43 of 115) of the mepolizumab arm and 20.2% (21 of 104) of the placebo arm met the three-component definition of clinical remission (OR 2.36, 95% CI 1.28–4.34; p=0.006) at week 52 (figure 3a). For the four-component definition, with an ACQ-5 score <1.5, 44.3% (51 of 115) of the mepolizumab arm *versus* 24.0% (25 of 104) of the placebo arm achieved clinical remission (OR 2.52, 95% CI 1.41–4.50; p=0.002). With an ACQ-5 score ≤0.75, 31.3% (36 of 115) of the mepolizumab arm *versus* 13.5% (14 of 104) of the placebo arm achieved clinical remission (OR 2.93, 95% CI 1.47–5.82; p=0.002) (figure 3b). In patients on high-dose ICS at baseline, similar clinical benefits in terms of an increased likelihood of achieving clinical remission were observed with mepolizumab *versus* placebo (figure 3 and supplementary table S1).

## Discussion

This study is the first RCT-based analysis to investigate the treatment effect of mepolizumab add-on *versus* a comparator in patients with SA-EP on medium-dose ICS. Mepolizumab led to greater clinical benefits *versus* placebo in this patient population across multiple outcomes, as evidenced by the larger reduction in the rate of CSEs, longer time to first CSE and first exacerbation requiring hospitalisation or ED visits, greater improvements in SGRQ score, ACQ-5 score and FEV_1_, and higher likelihood of clinical remission. Additionally, mepolizumab-treated patients on medium-dose ICS at baseline achieved a higher rate of clinical remission than those on high-dose ICS.

The advent of biologics like mepolizumab has significantly shifted asthma management from a “treat-to-failure” approach (escalating therapy to the maximum recommended dose when clinical improvement is not reached) to a “treat-to-target” strategy [3]. This new strategy targets key pathological pathways, tailoring treatments to individual needs and aiming for sustained disease remission or reduced disease activity [3]. The concept of clinical remission as a treatment goal has gained attention with the increasing use of biologic therapy for severe asthma.

This study demonstrated that mepolizumab resulted in a significantly higher rate of clinical remission *versus* placebo in patients on medium-dose ICS. The achievement of clinical remission, along with other clinical benefits that mepolizumab conferred to patients on medium-dose ICS, reinforces the pivotal role of biologics in the new asthma management paradigm. Notably, patients on medium-dose ICS from this study had achieved a considerably higher rate of clinical remission than those on high-dose ICS, regardless of the definition used for clinical remission or the ACQ-5 score cut-off. This could be because medium-dose ICS indicates less severe disease, making remission easier to achieve. This aligns with a study suggesting that less severe asthma has a higher chance of remission and with the GINA report, which outlines predictors of clinical remission that are indicative of lower disease severity [3, 15]. Although the reasons remain to be explored, these results suggest that the early use of mepolizumab in the treatment pathway could increase the chance of clinical remission, which encourages further research on the early integration of biologics into the asthma treatment pathway.

In this study, 44.3% of patients on baseline medium-dose ICS achieved clinical remission within 52 weeks of mepolizumab treatment, as defined by a four-component definition (OCS-free, no CSE, ACQ-5 score <1.5 and change from baseline in pre-bronchodilator FEV_1_ ≥0 mL). This finding aligns with a retrospective, real-world study of mepolizumab in patients with severe asthma, which used a similar but slightly different definition of remission and found that 52% of patients achieved clinical remission after 1 year of treatment [33]. Notably, the proportion of patients achieving clinical remission with mepolizumab increased from 52% in the first year to 73% in the third year [33]. This finding highlights the significant impact of treatment duration and the necessity of careful assessment before making any changes to the patients’ treatment for those who have not achieved remission after 1 year of treatment, as extended therapy may offer additional remission-related benefits [33]. Therefore, it may be expected that extending mepolizumab treatment beyond 52 weeks in this study could result in a higher proportion of patients achieving clinical remission.

ICS has long been a cornerstone in the pharmacological management of asthma. However, their high-dose use is constrained by several factors, including plateauing efficacy at high-dose levels and considerable inter-individual variability in response to ICS [16]. Recently, it has been revealed that for asthma patients with a high blood eosinophil count, escalation to high-dose ICS did not prove more effective in reducing the rate of exacerbations than maintaining medium-dose ICS [34]. Concerns over the side effects associated with high-dose ICS also limit its use [35]. These challenges highlight an urgent need for the continued shift to personalised medicine.

Clinical benefits that patients on medium-dose ICS received from mepolizumab in this study are consistent with those which they received from T2-targeted biologics in other studies [31, 36–39]. In SA-EP patients on medium-dose ICS at baseline from the real-world REALITI-A study, 24 months of mepolizumab treatment reduced the rate of CSEs by 80% compared with the pre-treatment period (0.67 *versus* 3.39 events·yr^−1^; rate ratio 0.20, 95% CI 0.14–0.28) [31]. In the subgroup of patients receiving medium-dose ICS at baseline from the phase 3 QUEST study, treatment with dupilumab reduced the rate of severe exacerbations by 51% *versus* placebo (0.344 *versus* 0.697 events·yr^−1^) over 52 weeks [36]. Furthermore, the BISE trial demonstrated that, in patients with mild to moderate asthma receiving low–medium-dose ICS alone or low-dose ICS plus long-acting β-2 agonist, treatment with benralizumab improved pre-bronchodilator FEV_1_*versus* placebo [39]. Taken together, the current study, coupled with other studies, provides strong evidence that the add-on of T2-targeted biologics such as mepolizumab is a promising alternative to escalation to high-dose ICS for patients with SA-EP who are receiving medium-dose ICS but require treatment escalation.

A key strength of the current analysis is the utilisation of RCT data to examine the treatment effects of mepolizumab in patients on medium-dose ICS, enabling a direct and unbiased comparison. As such, this analysis reinforces the understanding of the efficacy performance of mepolizumab in this patient population beyond what real-world studies have provided. The *post hoc* nature is the main limitation, indicating that the current analysis may not be appropriately powered. Therefore, the findings from this subgroup analysis should be interpreted with caution and warrant further validation in prospective studies.

In conclusion, mepolizumab add-on therapy conferred greater clinical benefits *versus* placebo across multiple clinical outcomes, including clinical remission, to Chinese patients with SA-EP on medium-dose ICS. In addition, mepolizumab-treated patients on medium-dose ICS achieved a considerably higher rate of clinical remission than those on high-dose ICS. The clinical benefits observed with mepolizumab are likely multifactorial and may reflect combined effects of mepolizumab on airway inflammation, airway remodelling and corticosteroid responsiveness, which warrant further investigation. These findings demonstrate the potential of mepolizumab as an effective treatment alternative when escalation to high-dose ICS is required in this patient population and encourage more research on the early use of biologics in asthma treatment.

## Data Availability

For requests for access to anonymised subject-level data, please contact the corresponding author.

## Supplementary material

**Please note:** supplementary material is not edited by the Editorial Office, and is uploaded as it has been supplied by the author. Supplementary material 01652-2025.SUPPLEMENT
