# Supplementary material for: Add-on mepolizumab in patients with severe asthma on medium-dose inhaled corticosteroids
Source: ERJ Open Res. 2026 Aug 17;12(4):01652-2025. doi: 10.1183/23120541.01652-2025 (PMC13478892; doi:10.1183/23120541.01652-2025)
Supplement: Supplementary file 1 [file 01652-2025.SUPPLEMENT.pdf]

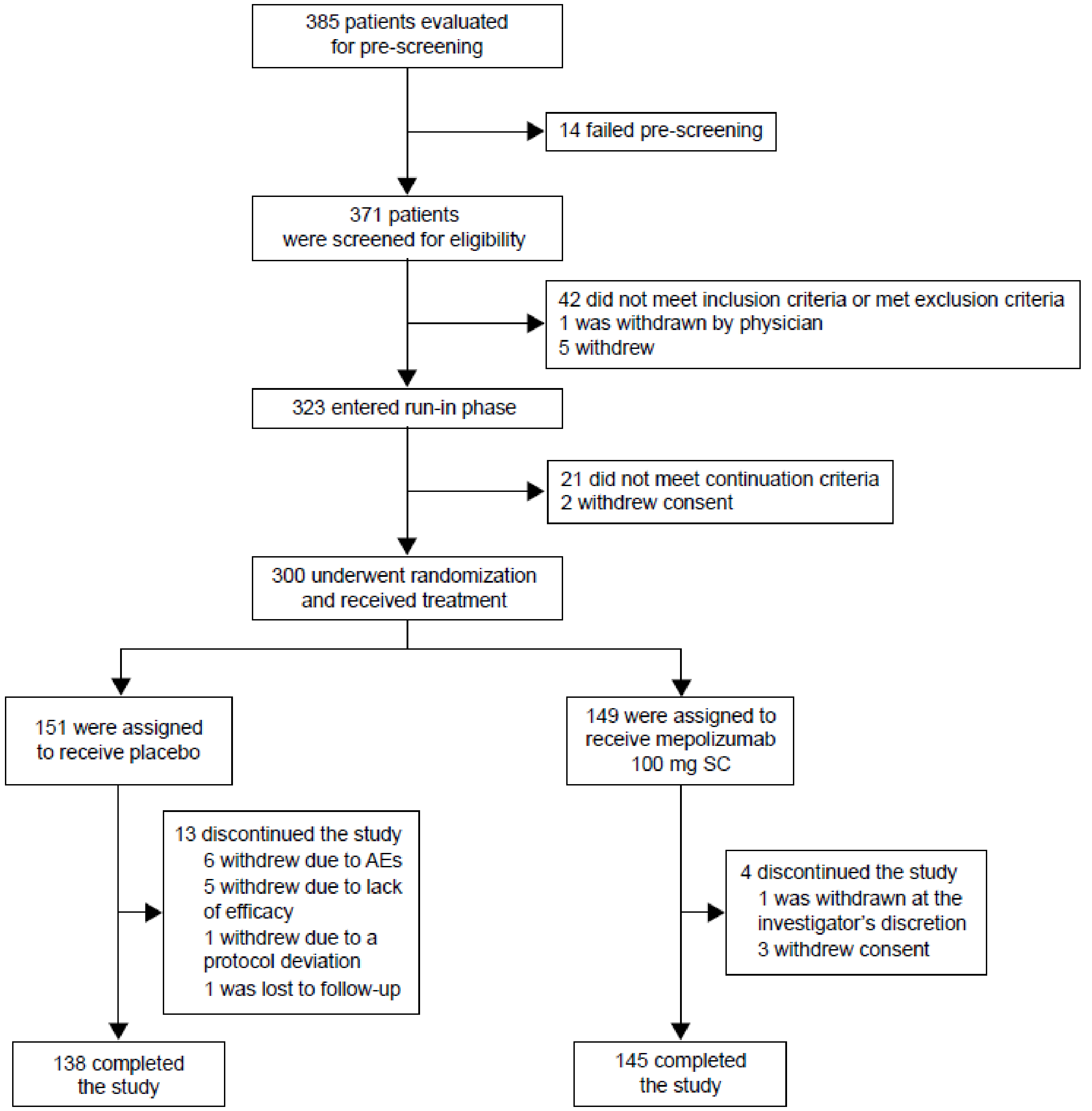

Supplementary Figure S1. CONSORT Diagram. Abbreviations: AEs, adverse events; SC, subcutaneously

Supplementary Table S1. Efficacy results in patients on high-dose ICS at baseline.

	Placebo (N=41)	Mepolizumab 100 mg SC (N=31)
**Annualised rate of CSEs**		
Exacerbation rate, events/year	1.94	0.46
Rate ratio (mepolizumab/placebo) (95% CI)	0.24 (0.12, 0.48)	
P-value	<0.001	
**Time to first CSE by Week 52**		
Probability of CSE (Kaplan-Meier estimate), %	67.5	33.7
HR (mepolizumab/placebo) (95% CI)	0.33 (0.16, 0.70)	
P-value	0.003	
**Time to first exacerbation requiring hospitalisation or ED visits by Week 52**		
Probability of CSE (Kaplan-Meier estimate), %	15.4	0.0
HR (mepolizumab/placebo) (95% CI)	0.00 (0.00, NE)	
P-value	0.994	
**SGRQ score at Week 52**		
Difference in least-square mean (mepolizumab vs. placebo)	-13.62	
95% CI	-22.57, -4.67	
P-value	0.004	
**ACQ-5 score at Week 52**		
Difference in least-square mean (mepolizumab vs. placebo)	-0.38	
95% CI	-0.78, 0.01	
P-value	0.055	
**Prebronchodilator FEV_1 at Week 52**		
Difference in least-square mean (mepolizumab vs. placebo), mL	241.29	
95% CI	19.24, 463.35	
P-value	0.034	
**Three-component definition of clinical remission with ACQ-5≤0.75**		
Proportion of patients achieving clinical remission, n (%)	4 (9.8)	8 (25.8)
OR (mepolizumab/placebo) (95% CI)	3.22 (0.87, 11.90)	
P-value	0.080	
**Three-component definition of clinical remission with ACQ-5<1.5**		
Proportion of patients achieving clinical remission, n (%)	8 (19.5)	14 (45.2)
OR (mepolizumab/placebo) (95% CI)	3.40 (1.19, 9.68)	
P-value	0.022	
**Four-component definition of clinical remission with ACQ-5≤0.75**		
Proportion of patients achieving clinical remission, n (%)	1 (2.4)	7 (22.6)
OR (mepolizumab/placebo) (95% CI)	11.66 (1.35, 100.69)	
P-value	0.025	
**Four-component definition of clinical remission with ACQ-5<1.5**		
Proportion of patients achieving clinical remission, n (%)	5 (12.2)	11 (35.5)
OR (mepolizumab/placebo) (95% CI)	3.96 (1.20, 13.02)	
P-value	0.023	

Abbreviations: ACQ-5, asthma control questionnaire-5; CI, confidence interval; CSE, clinically significant exacerbations; ED, emergency department; FEV_1, forced expiratory volume in one second; HR, hazard ratio; ICS, inhaled corticosteroid; OR, odds ratio; SC, subcutaneously; SGRQ, St George's Respiratory Questionnaire.
